# Spatio-temporal shifts in community structure and activity of *nirS*-type denitrifiers in the sediment cores of Pearl River Estuary

**DOI:** 10.1371/journal.pone.0231271

**Published:** 2020-04-21

**Authors:** Haitao Xie, Yiguo Hong, Huamin Liu, Lijing Jiao, Jiapeng Wu, Lixin Wang

**Affiliations:** 1 Key Laboratory for Water Quality and Conservation of the Pearl River Delta, Ministry of Education, Institute of Environmental Research at Greater Bay, Guangzhou University, Guangzhou, China; 2 The School of Ecology and Environment, Inner Mongolia University, Hohhot, China; Chinese Research Academy of Environmental Sciences, CHINA

## Abstract

Denitrification, an important process in microbial mediated nitrogen cycle, plays important roles in nitrogen loss in estuarine sediments. However, the function of denitrifiers in the estuarine subsurface sediments remained poorly understood. In this study, we analyzed the potential activity, abundance and community structure of *nirS*-type denitrifiers using ^15^N-labeled incubation quantitative-PCR and high throughput sequencing techniques in sediment cores from Pearl River Estuary (PRE). Results showed that subsurface sediments had nearly same level denitrification potential activity compare to surface sediments, although the abundance of *nirS* gene decreased sharply from surface to bottom in sediment cores. Meanwhile, *nirS* gene abundance exhibit significant temporal variations, which is consistent with denitrification potential activity. Moreover, the community structure and diversity of *nirS*-type denitrifiers in sediment cores exhibited remarkable temporal shift pattern. For spatial variation, no significant difference was observed of denitrifiers community structure in each sediment core from the surface to the subsurface, while there were significant different diversity characteristic among different cores. Redundancy analysis (RDA) showed that multiple environmental factors including salinity, pH, oxidation-reduction potential, nutrient content and organic substances synergistically shaped the diversity and distribution of *nirS*-type denitrifers in PRE sediments. Our results showed that *nirS*-type denitrifers played important roles in the nitrogen removal in subsurface sediments of PRE.

## Introduction

Microbe-mediated denitrification is a biogeochemical process, where nitrate (NO_3_^-^) is reduced stepwise to gaseous end-products, such as nitric oxide (NO), nitrous oxide (N_2_O) and nitrogen gas (N_2_) [1]. The produced gases are concomitantly released, causing a fixed nitrogen to be lost to the atmosphere [2]. These nitrous oxide greenhouse gases are significant contributors to global warming [3]. Denitrification is a redox process occurring only under anaerobic or low oxygen conditions [4–6], where NO_3_^-^ or NO_2_^-^ as the terminal electron acceptor produce NO, N_2_O and N_2_ through a sequence of electrochemical gradient and a series of oxidoreductases. Among them, nitrite reductase (NIR) is a key enzyme involved in denitrification, catalyzing the first key step that produces gaseous intermediates [7]. There are two kinds of structurally different but functionally equivalent NIR enzymes, containing cytochrome cd1 (NirS-type) or Cu (NirK-type), encoded by *nirS* and *nirK* gene, respectively [8–10]. Because denitrification is catalyzed by a variety of denitrifiers that taxonomically belong to different genera, diversity analysis using 16S rRNA gene may not be applicable to denitrifiers due to the relatively conservative sequence changes [11]. On the contrary, functional genes encoding nitrite reductase are characterized with more sequence changes, which can not only analyze the microbial community structure, but also identify the microbial communities’ ecological functions. The functional genes of *nirS* and *nirK* have been proven to be credible molecular markers to reveal the community composition and structure of denitrifying microbes [12], which have been widely used to investigated the diversity of denitrifers in various environments, such as the aquatic ecosystems, sediments and also the wastewater treatment plant (WWTP) [13–15]. Previous studies showed that *nirS*-type denitrifers make up more than 70% of known denitrifiers [16]. Therefore, *nirS* gene has been used as the universal molecular marker to explore the distribution and community structure of the denitrifying communities.

In previous studies, clone libraries combined with Sanger sequencing technology were usually employed to generate DNA sequences amplified from the microbial community. However, due to the high cost and low throughput, it remains difficult to deeply reveal the diversity of environmental microbes [17]. In recent years, high-throughput sequencing technology has been widely used to investigate microbial diversity. For example, Zhou *et al* [12] investigated the diversity and community structure of denitrifiers containing *nirS* gene in Zhoucun, and Fan *et al* [18] analyzed the ecological distribution of denitrifers in Lake Taihu reservoir using Illumina Miseq sequencing technology. However, a suitable reference taxonomic database of *nirS* gene has not yet to be constructed and the similarity threshold of *nirS* gene for OTU (operational taxonomic unit) clustering has also not yet been determined. These problems greatly affect diversity analysis to denitrifiers using high-throughput sequencing technology.

The Pearl River is the second largest river in China in terms the volume of freshwater discharge [19]. The Pearl River system consists of six main streams, including Xijiang, Beijiang and Dongjiang, as well as Zengjiang, liuxi and Tanjiang, where they converge downstream and eventually flow into the South China Sea [20–22]. In the past few decades, rapid industrialization and urbanization have led to an excessive release of pollutants into the estuary, intensifying eutrophication [23]. As the resuspending of organic nitrogen coupled with the diffusion of nitrate into the overlying water, the rate of denitrification in sediments have been usually significantly higher than those in other kinds of natural environments [24], making estuarine sediments into significant players in the removal of reactive nitrogen [2,25,26]. Specifically, surface sediments are known to be hot spots where fixed nitrogen get lost, reducing the anthropogenic nitrogen input and keeping balance of nitrogen budgets in estuarine ecosystem. However, the community structure of denitrifiers and its role on nitrogen loss remain poorly understood in the subsurface estuarine sediments. Here, we reported the activity, abundance, diversity, and community structuring of *nirS*-tpye denitrifiers in subsurface sediments of the Pearl River Estuary with high-throughput sequencing technology. Our findings highlight the importance of subsurface sediments as sinks for buried nitrogen in eutrophic estuarine ecosystems.

## Material and methods

### Study area and samples collection

The Pearl River Estuary (PRE) is a typical subtropical estuary, located in the south of China. It is a transition zone between freshwater and seawater adjacent to the South China Sea [27–29]. With rapid industrial development, this estuary has been subjected to biological, physical, and chemical interactions within the aquatic environments. The physiochemical properties of this estuary remarkably vary from the upstream to downstream section. Moreover, anthropogenic eutrophication in the PRE has become more and more serious in the past 30 years, influencing environmental health in this coastal region.

Sediment cores were collected from PRE in January and August 2016, using a gravity stainless steel sediment core sampler with a PVC tube (KC-Denmark) in five sites (PRE-1, PRE-3, PRE-7, PRE-13, and PRE-18) along the main stream. Detailed sampling sites are shown in S1 Fig. Sediment cores were sliced at 4-cm intervals from the surface to the bottom. The sediment samples were immediately placed in sealed polyethylene bags and stored at −20°C. Meanwhile, the sediment pore water from each sliced section was extracted by centrifugation at 5,000 rpm for 20 min (Eppendorf 5804R), and then filtered through 0.22 μM membrane for dissolved inorganic nitrogen analysis. pH and Oxidation-Reduction Potential (ORP) of sediments were recorded with a multi-parameter water quality analyzer (YSI 6600, USA) and ORP meter (Mettler-Toledo, Switzerland), respectively. Other environmental parameters including salinity, ratio of C/N, fixed ammonia (N_fix_), organic nitrogen (N_org_), total nitrogen (N_tot_) and organic carbon (C_org_) were also analyzed (S1 Table) as suggested previous methods [26].

### Preparation for ^15^N-labeled incubation experiment

The potential rates of both anammox and denitrification can be estimated using the quantities of ^29^N_2_ and ^30^N_2_ detected from MIMS following the methods of Risgaard‐Petersen *et al* [30] and Hou *et al* [31]. Briefly, slurry was prepared by combining the sediments and water volume at a ratio of 1:7 in 500 ml plastic bottle (Nalgene), and then transferred into a 12.5ml vial (Labcor), which was then sealed with rubber stoppers after being purged with helium for over 25 min and stirred evenly. In order to consume the residual NO_X_^-^, the vials were pre-incubated in the dark at 16°C (for winter samples)/30°C (for summer samples) for 24 h. Subsequently, these vials were spiked with helium-purged stock solution of ^15^NO_3_^-^. The final concentration of ^15^N in the mixture was at 100 μmol/L. After 8 h incubation, the incubation was poisoned by injecting 200 μL of 50% ZnCl_2_ solution into each to stop biological activity, and kept at room temperature in the dark until used for MIMS (Membrane Inlet Mass Spectrometer, HPR-40, US) [32].

### DNA extraction and PCR amplification

Total DNA was extracted from 0.25g sediments of each sample using the DNeasy® PowerSoil® Kit according to manufacturer’s instructions. The concentration and quality of the extracted DNA was determined using a NanoDrop™ Lite (Thermo Scientific, Wilmington, ME, USA). A specific primer pair cd3aF (5Ȳ-GTSAACGTSAAGGARACSGG-3Ȳ) and R3cd (5Ȳ-GASTTCGGRTGSGTCTTGA-3Ȳ) [33] was used to amplify *nirS* gene of the denitrifiers in the sediments of the PRE. PCR amplification was carried out in a reaction mixture of 25 μL containing 12.5 μL GoTaq Green Master PCR Mix, 0.5 μL of each primer, 10.5 μL nuclease-free water, and 1 μL template DNA. Then, PCR conditions included an initial denaturation at 95°C for 5 mins, followed by 33 cycles of 95°C for 45 s, 56°C for 1 min, 72°C for 1 min, and then final extension at 72°C for 10 mins. The forward primer cd3aF was attached to a unique 8 bp barcode sequence. All PCR products were extracted from agarose gel using MiniBEST Agarose Gel DNA Extraction Kit Ver.4.0 and the purified products were quantified using 2200 Tape Station and D1K Reagents. Finally, the PCR products containing targeted *nirS* gene were sequenced via high-throughput sequencing in GENEWIZ Company (Suzhou, China). Sequences were quality filtered and those with low base signals were removed, followed by the trimming of the barcode and primer sequences. Finally, quality-filtered sequences were retained and used for further analysis.

### Quantification of *nirS* gene from denitrifiers

*NirS* gene abundance in the sediments of the PRE was quantified using q-PCR using the Roche Lightcycler 480 Real Time PCR System. Targeted *nirS* gene were linked with pMD^®^ l8-T Vector kit (Takara, Dalian, China), and then converted to DH5α cell of *E*. *coli* to obtain standard plasmid with *nirS* gene after culturing. The plasmid DNA concentration was determined on a NanoDrop™ Lite (Thermo Scientific, Wilmington, ME, USA) and diluted 10-fold to generate standard curve. In a 15 uL reaction mixture, *q*PCR amplification was carried out in triplicate containing 7.5 μL Power SYBR Green qPCR Master Mix, 0.4 μL of each primer (cd3aF/R3cd), 5.7 μL of dd H_2_O, and 1.0 μL of template. PCR conditions included an initia denaturation at 95°C for 10 min, followed by 45 cycles of denaturation at 95°C for 15 s, annealing at 56°C for 45 s and extension at 72°C for 45 s. The melting curve method (fluorescence detection) was used to examine the specificity of PCR products, which was divided into three steps: denaturation at 95°C for 10 s, 65°C for 1 min and continuous collection of fluorescence signals at 95°C. The final step included cooling at 40°C for 10 s.

### Diversity analysis of *nirS* gene with high-throughput sequencing

*NirS* gene sequences were analyzed using MOTHU (V.1.35.1) with standard operating procedures (https://mothur.org/wiki/MiSeq_SOP) [41]. The selected sequences were clustered into operational taxonomic units (OTUs) at a 93% cut-off value. Rarefaction curves were generated to verify the effect of sequencing depth on the level of detected diversity in each sample. Alpha-diversity indices including ACE, Chao1, Shannon and Simpson index were estimated from the samples. The community structuring of *nirS*-type denitrifiers were visualized using principal coordinates analysis (PCoA) implemented in GraphPad Prism 7.00 software. Phylogenetic analysis of denitrifiers at the OTU level was performed in MEAG 7.0 software. The correlation between Shannon index, *nirS* gene abundance, and potential denitricication rates with environmental factors were determined by Pearson’s correlation in SPSS 24 Statistics (IBM, USA). Finally, the influence of environmental parameters on the denitrifiers community was investigated by redundancy analysis (RDA) using Canoco 5.0. The raw illumina sequencing reads of *nirS* gene were deposited in the NCBI short-read archive under the accession numbers PRJNA560762.

## Results

### Abundance of *nirS* gene and potential rate of denitrification in the sediments of the Pearl River Estuary

**Fig 1:**
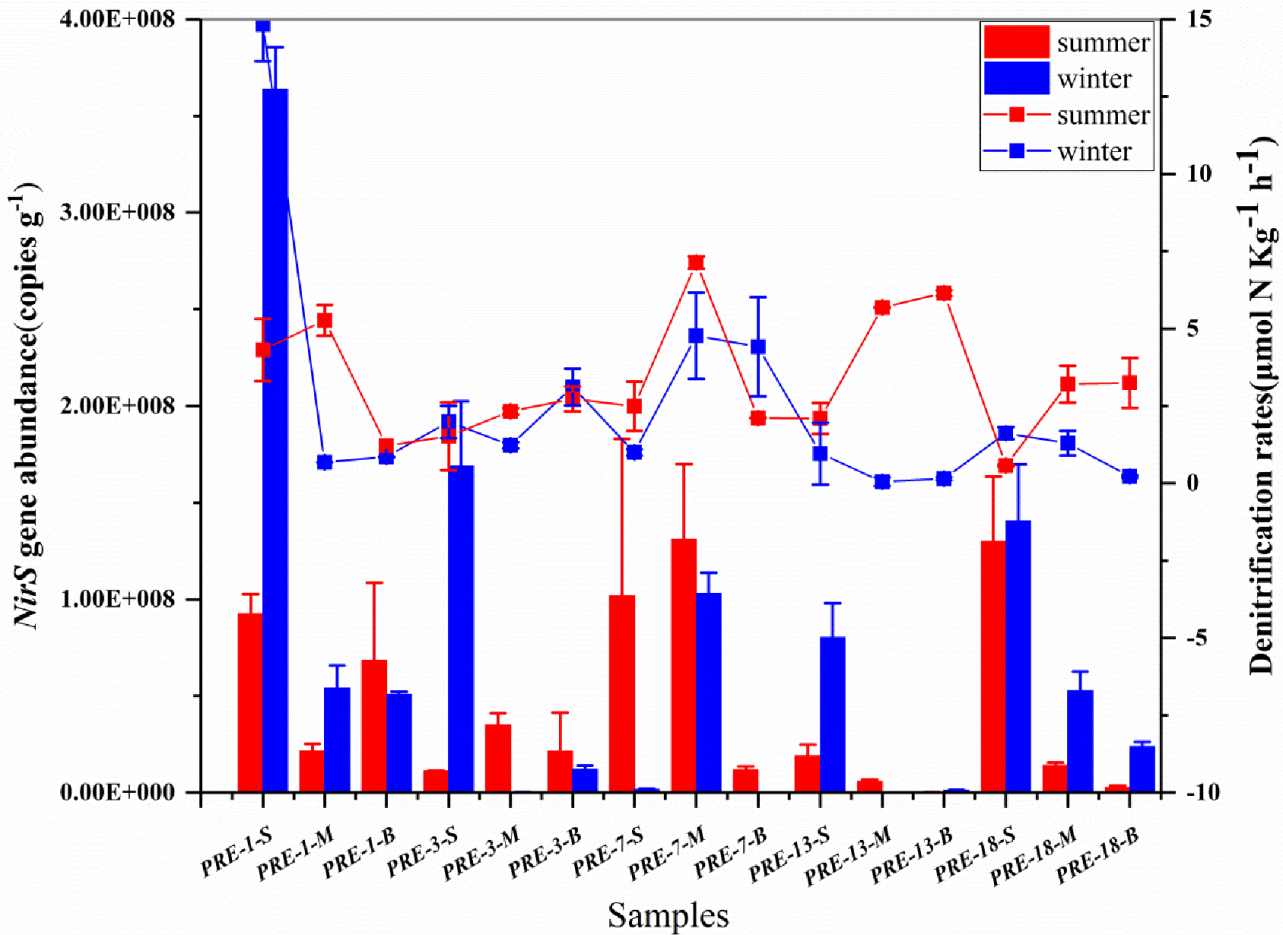
Abundances *nirS* gene (bar) and potential rates of denitrification (dot) in the sediments of Pearl River Estuary. Bars represent standard error based on three replicates.

As shown in Fig 1, the abundance of *nirS-*type bacteria in the sediments exhibited significant spatial and temporal variations. Vertically in each sediment core, the abundance of *nirS* gene decreased gradually from the surface to the bottom layer. The average values of abundances of *nirS*-gene were 1.11×10^8^, 4.64×10^7^ and 2.14×10^7^ copies g^-1^ in surface, middle and bottom sediment, respectively. However, no significant difference of *nirS* gene abundance was observed among five sediment cores from upstream to downstream, except the significant high value in the sample of PRE-1-WS. The abundance of the *nirS* gene was ranged from 2.52×10^5^ to 3.63×10^8^ copies g^-1^ in winter and from 1.70×10^5^ to 1.31×10^8^ copies g^-1^ in summer. The average abundance of *nirS* gene in winter was significantly higher than that in summer except in the sediment core 7, which had a opposite trend.

Although there was a significant difference in *nirS* gene abundances vertically in the cores, the potential rates were not different in significant except in the surface sediment core 1 (Fig 1). Interestingly, the denitrification potential rates in the subsurface sediments remained relatively high, reflecting an active loss of nitrogen via denitrification in the subsurface layers. In winter, the potential denitrification rates in the sediments ranged from 0.05±0.14 to 14.83±1.18 μmol N kg^-1^ h^-1^ with an average of 2.48 μmol N kg^-1^ h^-1^. In summer, the potential denitrification rates in the sediments ranged from 0.57±1.10 to 7.14±0.20 μmol N kg^-1^ h^-1^ with an average of 3.34 μmol N kg^-1^ h^-1^, and the highest rate (14.83±1.18 μmol N kg^-1^ h^-1^) was observed in the surface sediment of PRE-1 in winter. Moreover, the potential rates of anammox ranged from zero to 0.73±0.03 μmol N kg^-1^ h^-1^ with an average of 0.37 μmol N kg^-1^ h^-1^ in winter, and ranged from zero to 1.10±0.03 μmol N kg^-1^ h^-1^ with an average of 0.17 μmol N kg^-1^ h^-1^ in summer. Based on the potential rates of denitrification and anammox, the relative contributions of denitrification to nitrogen loss were 27.48~100% in winter and 34.18~100% in summer (S2 Table), suggesting that denitrification was a main process removing the nitrogen in the sediments of PRE.

### Diversity of *nirS*-type denitrifiers in PRE sediments

A total of 103,164 reads were obtained from 30 samples collected from sediments of PRE. High-quality sequences were obtained ranging from 1642 to 4551 sequences per sample (in average of 3289). The sequences were clustered at 93% similarity by MOTHUR software, a total of 9,038 OTUs were obtained through cluster analysis, and the final count of OTUs was 2,343 after removing the rare OTUs. The number of OTUs in each sample ranged from 228 to 729. Higher coverage (more than 93%) suggested that the OTUs of each *nirS-*type denitrifer library had been well captured. OTU counts were generally higher in the surface sediments than that in the subsurface sediments and the amount of OTUs also increased and then decreased from the upstream to downstream. Moreover, the average count of OTU was 601 in summer, significantly higher than in winter counted with 385 (Table 1).

**Table 1:** Alpha-diversity index of *nirS* gene sequences from PRE sediment cores on 7% dissimilarity level.

Samples	Nseqs	Coverage	OTUs	Ace	Chao1	Shannon	Simpson
PRE-1-WS	3942	0.96	482	662.52	663.18	4.11	0.11
PRE-1-WM	4415	0.97	297	436.21	432.72	3.29	0.12
PRE-1-WB	4551	0.97	273	521.70	406.06	2.32	0.38
PRE-3-WS	4245	0.97	325	473.84	476.44	3.55	0.14
PRE-3-WM	4051	0.98	276	346.73	341.55	3.76	0.07
PRE-3-WB	4434	0.98	228	461.85	365.33	2.31	0.37
PRE-7-WS	4043	0.93	638	1244.13	959.49	5.09	0.02
PRE-7-WM	3832	0.93	539	1146.56	905.74	4.74	0.03
PRE-7-WB	3737	0.96	418	614.80	600.18	4.33	0.04
PRE-13-WS	3020	0.93	478	1010.26	834.28	5.01	0.02
PRE-13-WM	1642	0.93	237	511.98	425.36	3.95	0.06
PRE-13-WB	3685	0.97	311	429.60	416.11	3.93	0.07
PRE-18-WS	3633	0.96	369	524.13	480.95	4.08	0.08
PRE-18-WM	3093	0.94	440	715.43	658.68	4.62	0.03
PRE-18-WB	3392	0.94	471	764.72	752.35	4.85	0.02
PRE-1-SS	3144	0.90	683	1285.04	1045.20	5.72	0.01
PRE-1-SM	3243	0.91	594	1265.55	924.00	5.28	0.01
PRE-1-SB	3142	0.91	613	1215.12	957.38	5.38	0.01
PRE-3-SS	2956	0.89	711	1361.23	1098.72	5.81	0.01
PRE-3-SM	3164	0.91	663	1064.26	988.82	5.64	0.01
PRE-3-SB	3249	0.92	617	1188.52	957.74	5.50	0.01
PRE-7-SS	2814	0.86	758	1773.10	1266.94	5.81	0.01
PRE-7-SM	2914	0.89	701	1101.44	1016.07	5.66	0.01
PRE-7-SB	3128	0.89	729	1581.82	1202.45	5.72	0.01
PRE-13-SS	3333	0.90	671	1529.13	1157.01	5.50	0.01
PRE-13-SM	3355	0.94	502	743.17	675.93	5.07	0.01
PRE-13-SB	3399	0.95	469	683.09	633.55	5.00	0.02
PRE-18-SS	3171	0.94	414	797.71	632.21	4.51	0.03
PRE-18-SM	3148	0.93	448	946.90	724.94	4.75	0.02
PRE-18-SB	3289	0.94	455	692.53	686.29	4.94	0.02

WS, WM and WB represent the surface sediments, the middle sediments and the bottom sediments in winter, respectively; SS, SM and SB represent the corresponding sampling sites while in summer.

To estimate community diversity and richness of *nirS*-type denitrifer in the sediments of PRE, the alpha diversity indexes of Chao1, ACE, Shannon, Simpson and Coverage indices were calculated (Table 1). The ACE and Chao1 diversity estimators for *nirS*-type denitrifer ranged from 346.73 to 1773.10 and 341.55 to 1266.94, respectively. ACE estimators showed remarkable higher than and Chao1 in most of samples, suggesting that more species richness may exist in the sediments. And this estimation was confirmed by the rarefaction analysis that rarefaction curves showed no plateaus in most samples, indicating the presence of much higher diversity than measured in the *nirS* gene reads (S1 Fig). Additionally, there was obvious change of both Chao1 and ACE indexes between summer and winter times, suggesting the diversity of *nirS*-type denitrifers in PRE had remarkably seasonal shift. The Shannon richness ranged from 2.31 to 5.81 and fluctuated around 0.67, suggesting that *nirS*-type denitrifer community was relatively even in the sediments.

### OTU-level α-and β-diversity of *nirS*-type denitrifiers

**Fig 2:**
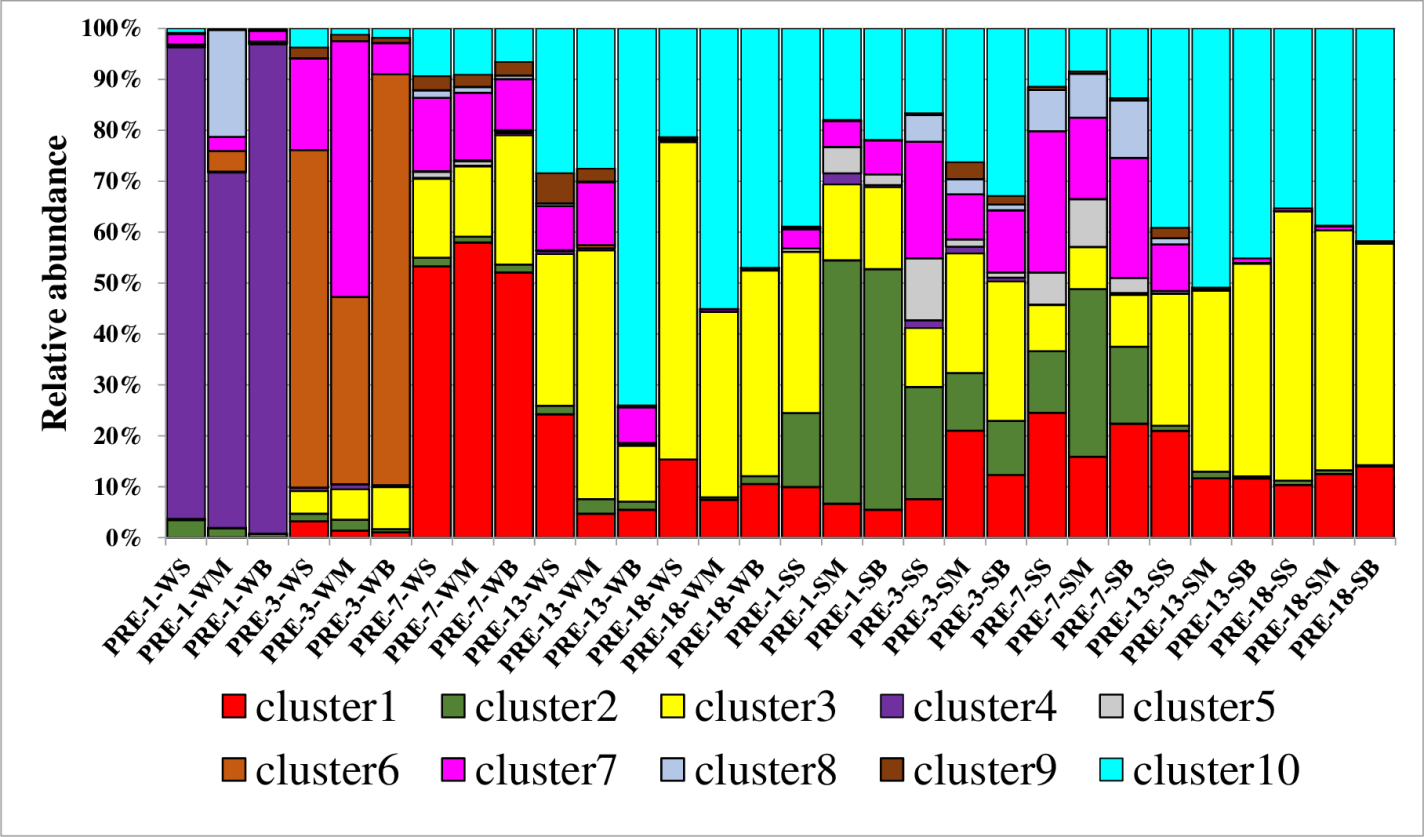
Relative abundance and taxonomic classifications of the denitrifiers retrieved from sediments of Pearl River Estuary at cluster level.

To better reflect the community diversity of denitrifiers, representative sequences of the top 50 OTUs were selected. As shown in S2 Fig, these top 50 OTUs phylogenetically grouped into 10 clusters. The amount of *nirS* gene sequences was highest in cluster 10, which accounted for 22.56% of the total sequences, while cluster 5 sequences only accounted for 0.84% of the total number of sequences. In the vertical direction from the surface to the bottom of sediment cores, the community composition of denitrifiers bacteria have no significant difference in summer and winter. On the contrary, the composition of *nirS*-type denitrifers have significant different in different station from upstream to downstream. In the PRE-1 sediments, cluster 4 was the dominant in the winter, but the community structure shifted into mutiple clusters in summer, including cluster1, cluster2, cluster3, cluster7 and cluster10. In the PRE-3 sediment, there were remarkable different kinds of *nirS*-type denitrfers, with the major group of cluster 6 and 7 in winter and cluster1, cluster2, cluster3, cluster7 and cluster10 in summer. In the PRE-7 sediments, there was also significant different community structure of *nirS*-type denitrfers in winter and summer, with the major group of cluster1, cluster3, cluster7 and cluster10 in winter and similar composition with PRE-3 in summer. However, in PRE-13 and PRE-18 sediment cores, the community of *nirS*-type denitrifers was made up of cluster1, cluster3 and cluster10 and no obvious change was observed (Fig 2). A heatmap analysis in OTUs level further showed a significant spatial shift and temporal pattern of the community of *nirS*-type denitrifers in PRE (S3 Fig).

**Fig 3:**
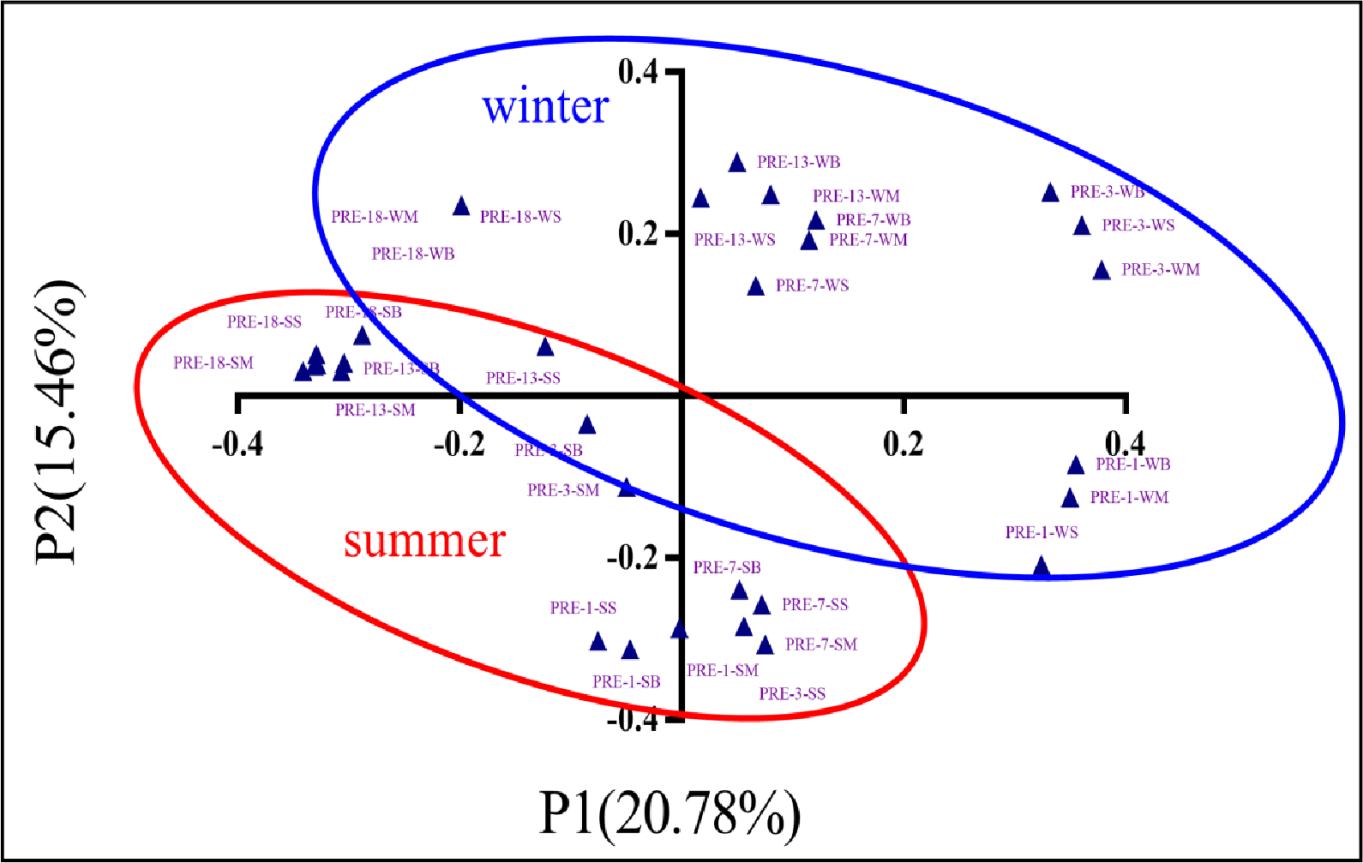
Principal coordinates analysis (PCoA) of *nirS* gene based on dominant OTUs in sediments of Pearl River Estuary. The blue circle represents the samples in winter and the red circle represents the samples in summer.

PCoA analysis presented an obvious spatio-temporal distribution of *nirS*-type denitrifers in the sediment cores of Pearl River estuary (Fig 3). The plots of the first two principal coordinate axes (P1 and P2) explicated 36.24% of the *nirS*-type denitrifers community variability among all the samples. The results showed that *nirS*-type denitrifers assemblage fell into two groups in summer and winter, indicating an apparent seasonal variance in the *nirS*-type denitrifiers in the PRE sediments. The sequences from each sediment core were all grouped together but formed a separated group, further confirmed the spatial pattern of the distribution of *nirS*-type denitrifiers in PRE sediments.

### Correlation between the environmental parameter and the distribution of *nirS*-type denitrifiers

**Fig 4:**
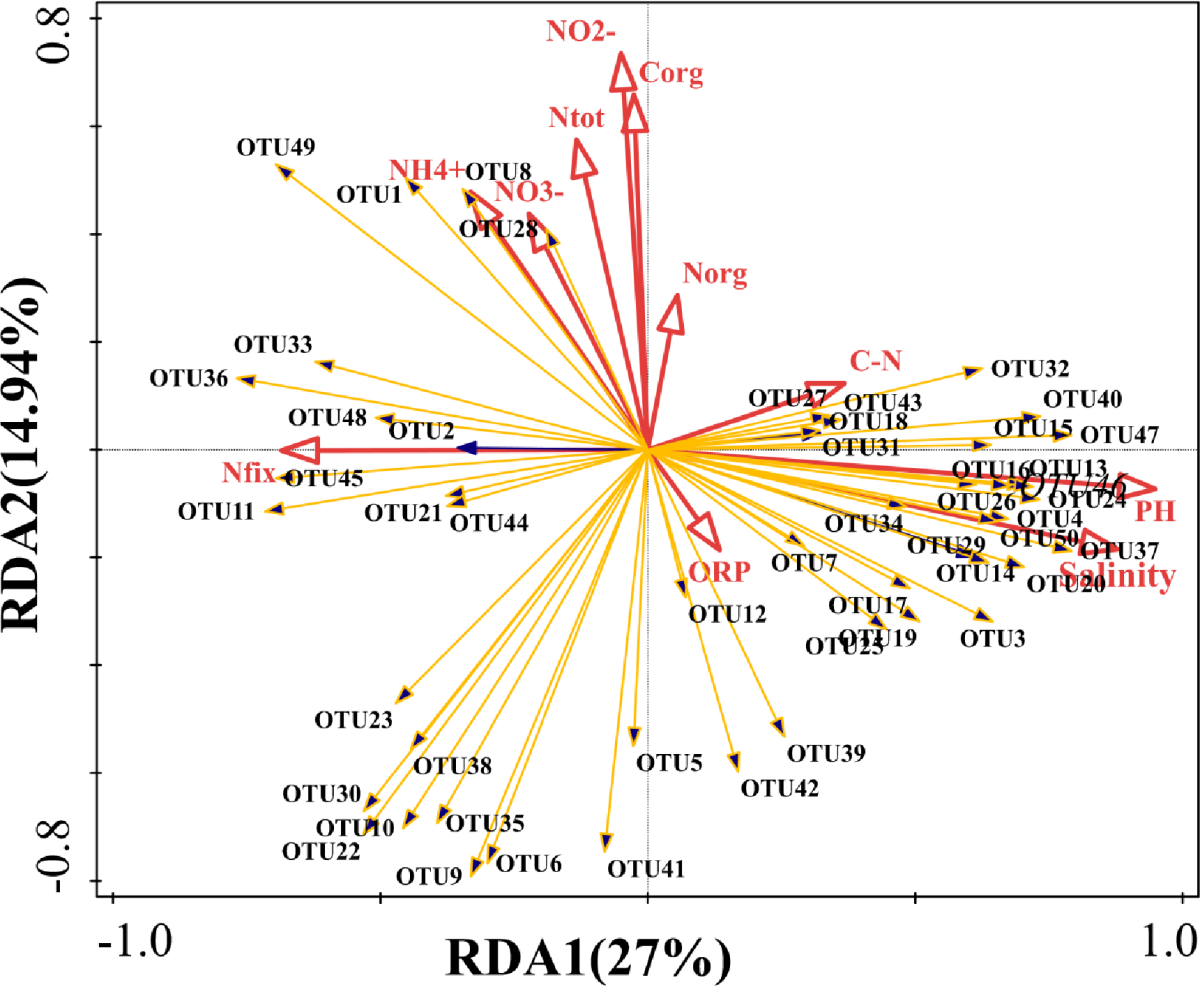
Redundancy analyses (RDA) of the communities and environmental parameters. Each yellow arrow represents an individual OTU and the red arrows represent statistically significant environment variables that explain the corelation patterns (P < 0.05).

The relationship between community structure and environmental factors were explored by Redundancy Analysis (RDA) (Fig 4), where the first two principle components explained 27% and 14.94% the community variation, respectively. Of all environmental parameters, salinity, exchangeable nitrogen (N_ex_), C_org_, N_org_, and C_org_:N_org_ were shown to be significant in driving the shift of community structure of *nirS*-type denitrifers. pH (*F* = 8.4, *P*<0.05) was the most significant environmental parameter influencing community structuring of the denitrifiers in the sediments of PRE, accounting for 24.5% of total variance. Correlation analysis between environmental factors with Shannon index, *nirS* gene abundance, and potential denitrification rates were determined using Pearson’s correlation (Table 2). Shannon index was significantly and positively correlated with salinity (*P*<0.01, *R* = 0.507), pH (*P*<0.01, *R* = 0.576) and ORP (*P*<0.05, *R* = 0.502), but negatively correlated with NH_4_^+^ (*P*<0.01, *R* = -0.683), N_tot_ (*P*<0.01, *R* = -0.540), N_org_ (*P*<0.05, *R* = -0.380) and C_org_ (*P*<0.01, *R* = -0.620). *NirS* gene abundance showed significantly positive correlation with NO_3_^-^ (*P*<0.01, *R* = 0.640) and NO_2_^-^ (*P*<0.01, *R* = 0.615), but negatively correlated with C/N (*P*<0.05, *R* = -0.395). Moreover, Pearson’s correlation values showed that potential rates of denitrifiers have significantly positive correlation with NO_3_^-^ (*P*<0.01, *R* = 0.603), NO_2_^-^ (*P*<0.01, *R* = 0.471), and *nirS* gene abundance (*P*<0.01, *R* = 0.545).

**Table 2:** The correlation analysis of environmental factors with Shannon index, *nirS* gene abundance and potential denitrification rates.

	Shannon index	Abundance	Denitrification rates
NO_3_^-^		0.640**	0.603**
NO_2_^-^		0.615**	0.471**
NH_4_^+^	-0.683**		
C-N		-0.395*	
pH	0.576**		
ORP	0.502**		
N_tot_	-0.540**		
N_org_	-0.380*		
C_org_	-0.620**		
Salinity	0.507**		

*.0.05 represents significant correlation;

**.0.01 represents extremely significant correlation.

## Discussion

In this study, the activity, abundance and community composition of denitrifying bacteria in sediments of PRE were analyzed deeply by high-throughput sequencing of *nirS* genes and isotope tracing method. The abundance of *nirS* gene ranged from 1.70×10^5^ to 3.63×10^8^ copies g^-1^ in the sediments of PRE, similar to those observed in an urban estuary in Jamaica Bay and in a subtropical estuary in Mexico, which had 5.2×10^5^ to 1.2×10^7^ copies g^-1^ and 2.72×10^6^ to 8.82×10^7^copies g^-1^, respectively [34,35]. At same time, the abundance of *nirS-*type denitrifiers exhibited spatial and seasonal shifts in sediments of PRE. Seasonal differences in *nirS*-type denitrifiers was apparent, having an average abundance of 8.10×10^7^ copies g^-1^ in winter, significantly higher than in summer (4.44×10^7^ copies g^-1^). The abundances of *nirS-*type denitrifiers in the subsurface sediments were significant lower than these in surface sediments, similar to the microbial distribution pattern reported in previous studies [36]. Most of heterotrophic microorganisms grow depend on decomposing organic matter. So, the abundance of microorganisms decreased with the increase of depth due to the change of organic matter in the sediment cores. Second, the community structure also exhibited significant spatial and temporal variations in the sediments cores of PRE. From upstream to downstream, the composition of *nirS*-type denitrifers shifted greatly, which should be resulted from the remarkable environmental change. Principal coordinates analysis (PCoA) also showed that community structure of the denitrifiers varied greatly and the *nirS* gene community clustered into distinct summer and winter groups. The distribute pattern of abundance and community structure of denitrifiers by encoding *nirS* gene were consistent with the previous studies of Gao *et al* in the coastal wetlands of China [37]. However, potential denitrification rates in the sediments did not appear apparent spatial shift. In terms of temporal variation, the average value is 3.34 μmol N kg^-1^ h^-1^ in summer, slightly lower in winter at 2.48 μmol N kg^-1^ h^-1^. No correlation between microbial activities and the abundance of *nirS* gene was found (P > 0.05), similar to the result observed by Zheng *et al* [38]. The mismatching of microbial activities (potential rates) and the abundance of *nirS* gene can be explained from two aspects. One hand, the potential rate does not represent the rate occurred in the real environment. Due the limitation of the substrates, the real denitrification rate in the subsurface would be lower than the observed one. On the other hand, the abundance determination was probably due to the limitations of DNA-based method. Some unknown microorganisms maybe not are captured by the primers used in this study. In addition to *nirS*-type denitrifers, *nirK-*type denitrifer was observed in estuarine systems [12,38,39,40]. Although we have done some investigation about *nirK* gene in the PRE, the primers for *nirK* gene need to be optimized. So, future studies should also investigate the *nirK* type denitrifiers compared to those *nirS* type. A future study analyzing from the RNA level could better illustrate the relationship between the quantity of microorganisms and their activities.

Correlation analyses showed that the potential denitrification rates were mainly affected by NO_3_^-^ (*P*<0.01, *R* = 0.603) and NO_2_^-^ (*P*<0.01, *R* = 0.471) (Table 2), suggesting that the NO_3_^-^ and NO_2_^-^ may be the main drivers of denitrification activities in the sediments of PRE. The high NO_3_^-^ concentration in the surface sediments and decreased sharply from the surface to subsurface sediments, supporting our hypothesis that denitrification would be very active in the subsurface sediments. Compared to NO_3_^-^, the concentration of NO_2_^-^ was very low in aerobic surface sediments, indicating that strong nitrification was occurring through aerobic microbial activity while denitrification was inhibited, eventually resulting in the accumulation of NO_3_^-^. In our investigation, NH_4_^+^ (*P*<0.01, *R* = -0.683) affected distribution of community diversity and exhibited significant negative correlation (Table 2). Indeed, in the sediment-water interface, the disturbance or even destruction of surface sediments by organisms increased the flux of ammonia nitrogen by breaking down and converting organic matter. Simultaneously, organic matter may be slowly mineralize and leading to the accumulation of ammonia nitrogen under anaerobic conditions in sediment cores, which also supported by the low NH_4_^+^ concentration in the surface sediments. Thus, N_org_ (*P*<0.01, *R* = -0.380) and C_org_ (*P*<0.01, *R* = -0.620) were significantly and negatively correlated with community diversity distribution in sediments of PRE. In contrast, at the bottom of the sediments, insufficient substrates reduced microbial metabolic activities, which also led a decrease in the diversity of denitrifiers [41]. Statistical showed that salinity was an important environmental factor affecting denitrifier community structure (Fig 4), similar to previous studies in different salinity habitats [15, 37, 38].

Compare to the denitrification activity, anammox activities were much lower. Anammox contributed 1% to 26% nitrogen loss in PRE sediments, so denitrification should be dominant microbial process for nitrogen loss in the PRE, consistent with previous studies in estuarine ecosystems [2,38,42,43]. Several studies have shown that surface sediment in estuarine ecosystem was an active zone due to its interactions with the overlaying water, leading to the frequent transport of reactive nitrogen, aerobic mineralization of organic matter, and the occurrence of various biogeochemical processes mediated by microorganisms [44,45]. Differences in the potential rates of denitrification with seasons and locations have already been reported in other ecosystems including wetlands [46, 47], soil [48], river [49], but remain poorly understood in estuarine ecosystems. Our study showed that the denitrification potential rates in the sediment cores did not exhibit distinct differences from the surface to the subsurface layer, with average values of 3.13 N kg^-1^ h^-1^ in the surface sediments, 3.17 N kg^-1^ h^-1^ in the middle fraction and 2.42 N kg^-1^ h^-1^ in the bottom sediments. These results suggest that the subsurface sediments should be played an important role in the nitrogen removal in PRE sediments and preventing eutrophication of estuarine ecosystem.

## Conclusion

This study showed the community structure and abundance of denitrifiers exhibited a strong spatio-temporal variance, but denitrification potential rates did not fluctuate greatly. Statistics also revealed that NO_3_^-^ and NO_2_^-^ were crucial factors influencing denitrifiers abundance and potential rates in subsurface sediments. Compare to the anammox, denitrification was the dominant microbial process for nitrogen loss in the PRE sediments. Furthermore, our study showed that the denitrification potential rates in the sediment cores did not exhibit distinct differences from the surface to the subsurface layer, indicating subsurface sediments played an important role in the nitrogen removal in Pearl River sediments.

## Supporting information

S1 Table Physical and chemical parameters in the sediments from PRE. Chemical parameters include the concentration of total nitrogen (N_total_), NH+ 4, NO- 2, NO- 3, organic nitrogen (N_org_), organic carbon (C_org_), and the ratio of C_org_:N_org_. Physical parameter include the oxidation reduction potential (ORP) and salinity.
(DOCX)

S2 Table The potential rates and relative contribution of anammox and denitrification in sediments of Pearl River Estuary. (DOCX)

S1 Fig Rarefaction curve of *nirS* gene in the sediments of the Pearl River Estuary. (DOCX)

S2 Fig Neighbor-joining phylogenetic tree of representative sequences of the top 50 OTUs. (DOCX)

S3 Fig A neighbor-joining phylogenetic tree and heatmap of the top 50 OTUs of denitrifiers in the sediments of the Pearl River Estuary. (DOCX)
