# Supplementary material for: Spatio-temporal shifts in community structure and activity of nirS-type denitrifiers in the sediment cores of Pearl River Estuary
Source: PLoS One. 2020 Apr 21;15(4):e0231271. doi: 10.1371/journal.pone.0231271 (PMC7173864; doi:10.1371/journal.pone.0231271)
Supplement: S1 Table — Chemical parameters include the concentration of total nitrogen (Ntotal), NH+ 4, NO- 2, NO- 3, organic nitrogen (Norg), organic carbon (Corg), and the ratio of Corg:Norg. Physical parameter include the oxidation reduction potential (ORP) and salinity. (DOCX) [file pone.0231271.s001.docx]

**Table S1. Physical and chemical parameters in the sediments from PRE. Chemical parameters** include the concentration of total nitrogen (N_total_), NH+ 4, NO- 2, NO- 3, organic nitrogen (N_org_), organic carbon (C_org_), and the ratio of C_org_:N_org_. Physical parameter include the oxidation reduction potential (ORP) and salinity.

| Samples | N_total_  (mg·kg^-1^) | NO_3_^-^  (mg·kg^-1^) | NO_2_^-^  (mg·kg^-1^) | NH_4_^+^  (mg·kg^-1^) | N_org_  (mg·kg^-1^) | C_org_  % | C_org_:N_org_ | ORP  mV | Salinity  % |
| --- | --- | --- | --- | --- | --- | --- | --- | --- | --- |
| PRE1S | 1169.16 | 34.76 | 4.17 | 14.02 | 973.68 | 1.31 | 11.28 | -179.67 | 0 |
| PRE1M | 962.13 | 27.23 | 1.75 | 31.90 | 751.52 | 1.24 | 12.95 | -179.75 | 0 |
| PRE1B | 1809.25 | 2.36 | 1.01 | 134.91 | 1511.4 | 2.17 | 12.01 | -138.25 | 0 |
| PRE3S | 1042.43 | 8.11 | 1.08 | 13.50 | 872.15 | 1.14 | 10.88 | -167.67 | 1.05 |
| PRE3M | 875.92 | 7.86 | 0.97 | 36.86 | 683.42 | 0.96 | 11.01 | -172.67 | 2.00 |
| PRE3B | 536.47 | 7.61 | 0.75 | 21.35 | 414.26 | 0.76 | 14.13 | -121.67 | 3.18 |
| PRE7S | 763.61 | 9.80 | 0.63 | 10.41 | 604.54 | 0.86 | 11.39 | -69.80 | 4.58 |
| PRE7M | 729.02 | 4.82 | 0.63 | 11.99 | 593.71 | 0.84 | 11.51 | -115.20 | 10.00 |
| PRE7B | 660.06 | 3.14 | 1.00 | 9.12 | 443.01 | 0.72 | 10.96 | -129.00 | 8.50 |
| PRE13S | 1068.40 | 5.35 | 0.53 | 5.26 | 944.49 | 1.24 | 11.59 | -125.00 | 7.96 |
| PRE13M | 1097.91 | 3.87 | 0.84 | 6.23 | 978.52 | 1.34 | 12.25 | -170.33 | 15.00 |
| PRE13B | 1057.48 | 7.40 | 0.13 | 9.32 | 902.19 | 1.23 | 11.66 | -166.67 | 13.87 |
| PRE18S | 892.34 | 5.92 | 1.75 | 15.17 | 738.65 | 1.05 | 11.62 | -122.67 | 18.47 |
| PRE18M | 761.89 | 2.40 | 0.71 | 5.14 | 640.93 | 1.00 | 13.17 | -126.00 | 26.72 |
| PRE18B | 594.87 | 2.07 | 0.33 | 5.27 | 460.70 | 0.84 | 14.05 | -133.67 | 29.48 |
