# Supplementary material for: Spatio-temporal shifts in community structure and activity of nirS-type denitrifiers in the sediment cores of Pearl River Estuary
Source: PLoS One. 2020 Apr 21;15(4):e0231271. doi: 10.1371/journal.pone.0231271 (PMC7173864; doi:10.1371/journal.pone.0231271)
Supplement: S2 Table — (DOCX) [file pone.0231271.s002.docx]

**Table. S2.** The potential rates and relative contribution of anammox and denitrification in sediments of Pearl River estuary.

|  |  | Denitrification | Anammox |
| --- | --- | --- | --- |
| Rates(μmol N Kg^-1^ h^-1^) | winter | 0.05 ± 0.14 to 14.83 ± 1.18 | 0.00 ± 0.01 to 0.73 ± 0.03 |
|  | summer | 0.57 ± 1.10 to 7.14 ± 0.20 | 0.00 ± 0.01 to 1.10 ± 0.03 |
| Contribution(%) | winter | 27.48 - 100 | 0 - 72.52 |
|  | summer | 34.18 - 100 | 0 - 65.82 |
