# Supplementary material for: Spatio-temporal shifts in community structure and activity of nirS-type denitrifiers in the sediment cores of Pearl River Estuary
Source: PLoS One. 2020 Apr 21;15(4):e0231271. doi: 10.1371/journal.pone.0231271 (PMC7173864; doi:10.1371/journal.pone.0231271)
Supplement: S1 Fig — (DOCX) [file pone.0231271.s003.docx]

**Fig.S1.** Rarefaction curve of *nirS* gene in the sediments of the Pearl River estuary
