# Supplementary material for: Spatio-temporal shifts in community structure and activity of nirS-type denitrifiers in the sediment cores of Pearl River Estuary
Source: PLoS One. 2020 Apr 21;15(4):e0231271. doi: 10.1371/journal.pone.0231271 (PMC7173864; doi:10.1371/journal.pone.0231271)

**Fig. S3**. A neighbor-joining phylogenetic tree and heatmap of the top 50 OTUs of denitrifiers in the sediments of the Pearl River estuary.


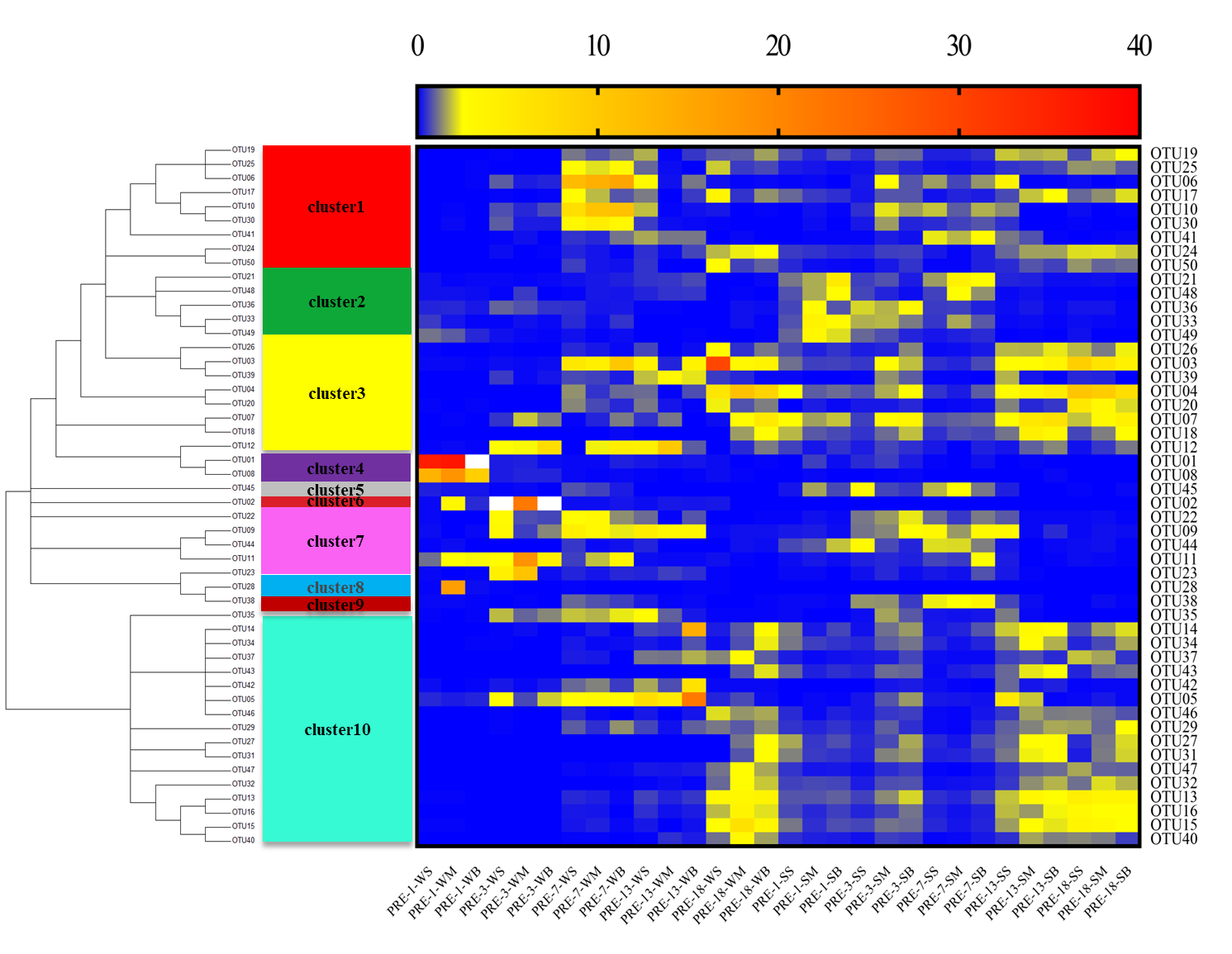

Supplement: S3 Fig — (DOCX) [file pone.0231271.s005.docx]
